# A systematic review of phenytoin intoxication induced by compound phenytoin sodium, ephedrine hydrochloride and theophylline tablets in China

**DOI:** 10.1097/MD.0000000000013689

**Published:** 2018-12-21

**Authors:** Lingxia Zhang, Zhenfei Li, Gaiying Ma, Xu Han, Cancan Li, Mingyue Shan, Liping Chen

**Affiliations:** Department of Neurology, The Second Hospital of Hebei Medical University, Shijiazhuang, Hebei, China.

**Keywords:** CPEHTT, nervous system, phenytoin poisoning, systematic review

## Abstract

**Objective:**

In this study, we aimed to review the literature on phenytoin intoxication induced by compound phenytoin sodium, ephedrine hydrochloride and theophylline tablets (CPEHTT).

**Method:**

A literature search was performed in the following databases: WANFANG DATA, HowNet, National Library Reference and Consultation Alliance, Full-text Database of Foreign Medical Journals, PubMed and Ovid. The search terms were “Compound Phenytoin Sodium, ephedrine Hydrochloride and Theophylline Tablets,” and “poisoning,” or “toxicity,” in Chinese and in English.

**Result:**

Ten articles including 104 patients with CPEHTT intoxication were identified. The ages of the patients ranged from 52 to 82 years. Sixty-seven patients were male and thirty-seven patients were female (the male/female ratio, approximately 2:1). The most common clinical manifestations were dizziness (85%) and ataxia (85%), followed by limb weakness (65%), diplopia (25%), binocular horizontal nystagmus (24%), limb numbness (13%), nausea and vomiting (12%), somnolence (10%), tremor and high muscle tension (7%), lag in response (5%), dysarthria (6%), choking cough (2%), auditory hallucination and visual fantasy (1%), and involuntary movement (1%). All patients had chronic lung disease, and the most common disease was chronic bronchitis. The dosage ranged 4 to 15 tablets per day with medication duration of more than 1 year for most patients.

**Conclusion:**

The CPEHTT intoxication caused by phenytoin toxicity represents a drug safety problem in China. The common clinical manifestations, serum phenytoin concentrations, and associated factors of CPEHTT intoxication are important for diagnosis and prevention. These findings may help guide clinicians to correctly attend to the use of CPEHTT and avoid its toxicity.

## 1 Introduction

Compound phenytoin sodium, ephedrine hydrochloride and theophylline tablets (CPEHTT) are a commonly used prescription drug for the treatment of bronchial spasms induced by bronchial asthma and chronic asthmatic bronchitis. The compound is widely used in China due to its therapeutic effectiveness and availability.^[1]^ However, adherence with the proper regimen is poor, especially among elderly patients who lack guidance from a professional physician or pharmacist, and during disease exacerbations. Patients often misuse the medication by independently adjusting their dosage, using it for prolonged periods, and overdosing.^[1]^ Since the therapeutic concentration (10–20 μg/ml) and toxic concentration (20 μg/ml and above) of phenytoin are very close,^[2]^ the safety margin of phenytoin is narrow. Thus, it is necessary to monitor the concentration of phenytoin in patients who use CPEHTT over prolonged periods.^[3]^ Patients with phenytoin toxicity often present with non-specific signs and symptoms that are similar to other neurological disorders, especially cerebrovascular diseases.^[4]^

In searching the literature, we identified many Chinese articles about CPEHTT toxicity. However, to date, there are no English articles about CPEHTT toxicity in the literature, suggesting that CPEHTT toxicity may represent a unique problem in China. Therefore, in this study, we performed a systemic review to summarize the cases of CPEHTT administration with phenytoin toxicity. We summarize the common clinical manifestations, underlying mechanisms, associated factors, treatment, and prognosis of CPEHTT intoxication. This article provides the basis for improving clinical diagnosis and treatment of CPEHTT intoxication.

## 2 Materials and methods

### 2.1 Literature inclusion criteria

Articles that met the following conditions were included in this study: Patients were treated with CPEHTT; CPEHTT intoxication was caused by phenytoin; and The clinical record for diagnosis and treatment or case characteristics of patients was complete. This study uses meta-analysis to provide a systematic review of phenytoin intoxication. Ethical review was not applicable.

### 2.2 Literature exclusion criteria

Articles that met the following conditions were excluded from this study: repeatedly published literature or case data; phenytoin poisoning was not attributed to CPEHTT; or the study objective was determining drug composition or the pharmacokinetics of CPEHTT.

### 2.3 Search strategy

#### 2.3.1 Database

The literature search was conducted using the following databases: WANFANG DATA, CNKI, National Library Reference and Consultation Alliance, Full-text Database of Foreign Medical Journals, PubMed and Ovid up to January 2017.

#### 2.3.2 Search strategy

The following terms were used: “Compound Phenytoin Sodium, Ephedrine Hydrochloride and Theophylline Tablets” and “poisoning” in Chinese; and “Compound Phenytoin Sodium, Ephedrine Hydrochloride and Theophylline Tablets”, and “poisoning” or “toxicity” in English. The language of the publication was not limited.

#### 2.3.3 Access to the original and data

The full text of each relevant article was retrieved based on the title or abstract of the article or by a manual search in the National Library Reference and Consultation Alliance.

## 3 Results

### 3.1 Characteristics of the reported literature

**Figure 1:**
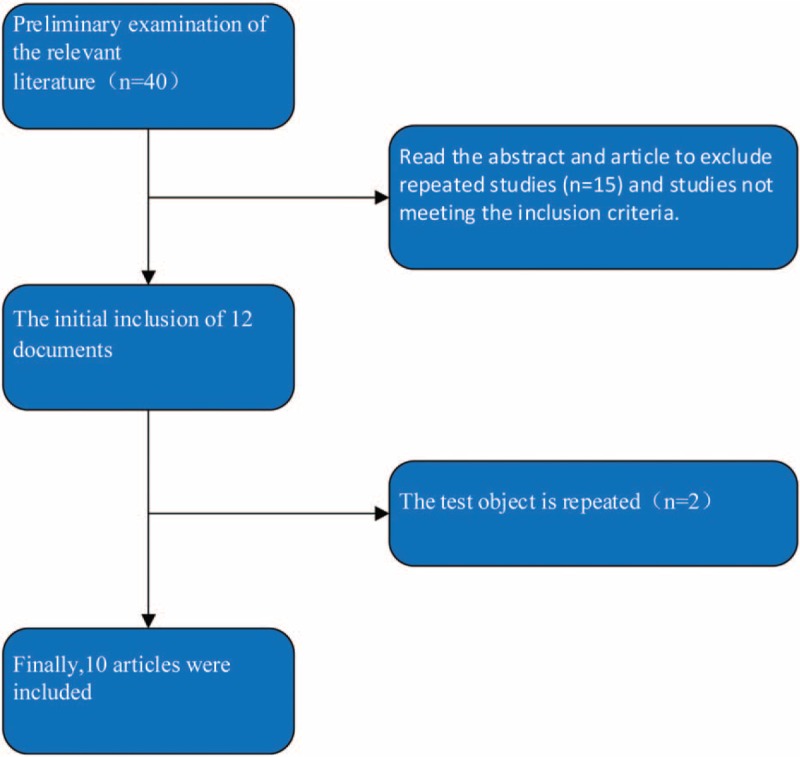
Literature search and screening process.

The initial literature search identified 12 articles that met the inclusion criteria. Two articles were excluded because they included repeated subjects. Finally, 10 articles were included in this review^[1,3–11]^ (Fig. 1). These articles included a total of 104 patients with CPEHTT intoxication. All the articles were published in Chinese journals between 2009 and 2016. Details of the included studies are summarized in Table 1.

**Table 1:**
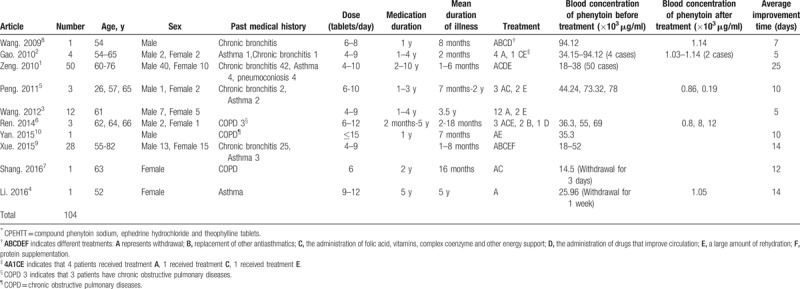
Summary of the literature on CPEHTT^∗^ toxicity.

### 3.2 Common clinical manifestations of CPEHTT intoxication

Table 2 summarizes the clinical manifestations of the 104 patients with CPEHTT intoxication. Of the 104 patients who were diagnosed with phenytoin intoxication induced by CPEHTT, the most frequent clinical manifestations were dizziness (n = 88, 85%) and ataxia (n = 88, 85%) including gait instability, adiadochokinesia, and abnormal finger-to-nose test followed by limb weakness (n = 69, 65%), diplopia (n = 26, 25%), and binocular horizontal nystagmus (n = 25, 24%). Other rare symptoms included limb numbness (n = 14, 13%), nausea and vomiting (n = 12, 12%), somnolence (n = 10,10%), tremor, increased muscle tone (n = 7, 7%), lag in response (n = 5, 5%), dysarthria (n = 6, 6%), choking cough during drinking (n = 2, 2%), auditory hallucination and visual fantasy (n = 1, 1%), and involuntary movement (n = 1, 1%).

**Table 2:**
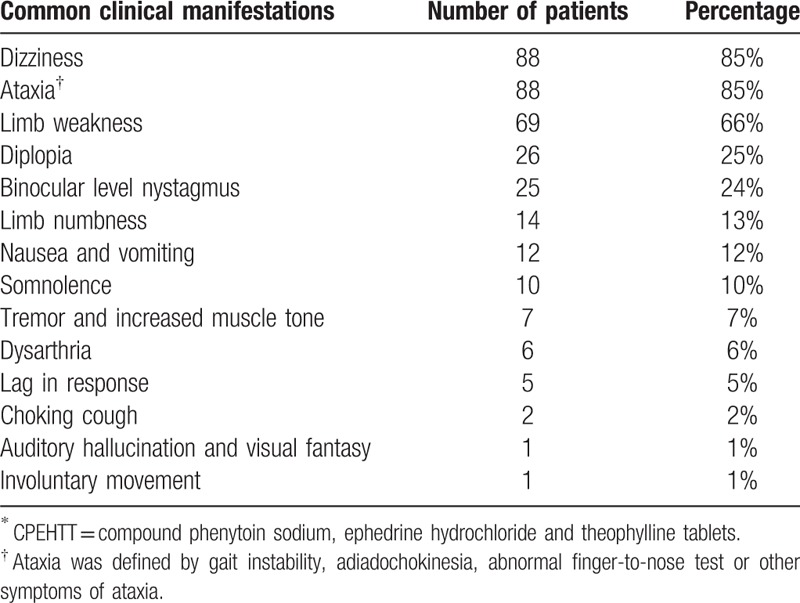
Frequency of different signs and symptoms in 104 patients with CPEHTT^∗^ poisoning.

### 3.3 Factors related to CPEHTT intoxication

#### 3.3.1 Age

With increased age, the maximum metabolic rate constant (Vm) of phenytoin decreases gradually. A reduction of blood flow and drug metabolic microsomal enzyme in the liver contributes to the age-related decrease in the Vm of phenytoin. Therefore, elderly patients are more prone to phenytoin intoxication.^[12]^ Of the 104 patients with CPEHTT intoxication, all were older than 52 years except one 26-year-old patient (Table 1). Therefore, elderly patients are more susceptible to CPEHTT intoxication.

#### 3.3.2 Sex

It has been reported that phenytoin poisoning occurs more commonly in men than in women (66.67% vs. 33.33%, respectively).^[13]^ Consistent with this finding, we found that CPEHTT intoxication occurred in 67 (64%) men and 37 (36%) women (Table 1). The male/female ratio of CPEHTT intoxication is approximately 2:1. Therefore, CPEHTT intoxication is gender-related, occurring more commonly in males.

#### 3.3.3 Dosage

The recommended dose for CPEHTT is 2 tablets 2 to 3 times a day after meals. After a week or symptoms are controlled, the dose is reduced to 1 to 2 tablets 1 to 2 times a day. The maximal dosage should not exceed 6 tablets at a time, and 10 tablets per day. Of the 104 patients with CPEHTT intoxication, most patients took 4 to 15 tablets per day. The exact relationship between intoxication and the dosage could not be determined due to the lack of detailed data in some studies. It has been reported that phenytoin poisoning is not entirely due to excessive dose,^[14]^ and the association between the daily dose and the presence of toxic reactions varies individually.^[13]^ However, excessive dosing is the most common cause of phenytoin poisoning.^[14]^ Therefore, CPEHTT intoxication is associated with medication dosage.

#### 3.3.4 Medication duration

Except in 2 cases where the patients took CPEHTT for 2 months and 6 months, most patients took the medication for more than 1 year, with the longest medication duration of 10 years. The patients did not reduce the amount after administration of CPEHTT for 1 week, but increased the dosage of the drug when symptoms were aggravated. Long-term use and overdose of phenytoin sodium can cause acute and chronic poisoning. It has been reported that cerebellar atrophy was more severe in patients with long-term use of phenytoin,^[15]^ and short-term toxic effects of phenytoin on the cerebellum were generally reversible.^[10]^ In addition, several studies have shown that even long-term normal doses or single overdoses of phenytoin can induce irreversible cerebellar atrophy.^[16,17]^ Therefore, CPEHTT intoxication is positively associated with medication time.

#### 3.3.5 Other factors

The CPEHTT poisoning may be related to liver and kidney dysfunction, malnutrition, and drug interactions. In this review, we found that only a few patients had slightly elevated transaminase and urea nitrogen in 2 of the 10 articles.^[3,8]^ In 2 articles, hypoglycemia^[1]^ or anemia^[1,10]^ were associated with poor therapeutic effect. Abnormal liver function results in slow metabolism, prolonged half-life, and accumulation of phenytoin, leading to toxicity. Renal dysfunction and hypoproteinemia also contribute to phenytoin toxicity, because decreased binding of phenytoin to plasma protein results in an increased concentration of free phenytoin in the plasma, leading to toxicity.^[12]^

### 3.4 Past medical history

**Figure 2:**
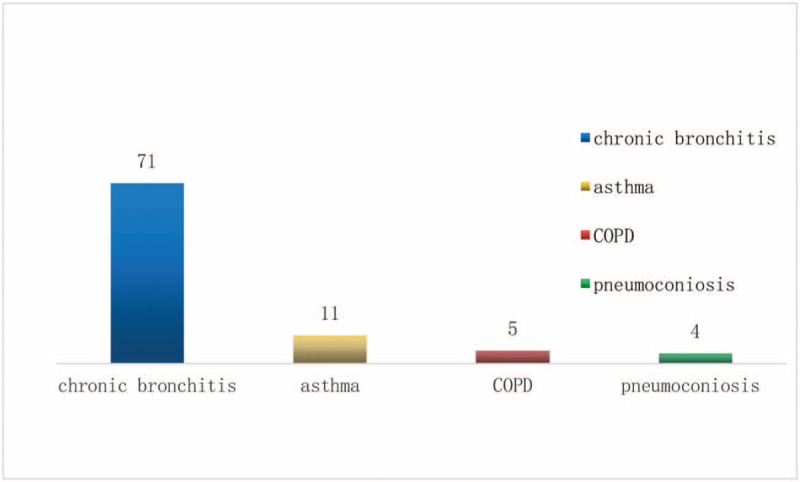
Distribution of types of lung disease among 104 study subjects.

Chronic lung disease is the main indication for CPEHTT. Of the 104 patients, 71 (78%) had chronic bronchitis, 11 (12%) had asthma, 5 (6%) had chronic obstructive pulmonary disease, and 4 (4%) had pneumoconiosis (Fig. 2). Therefore, CPEHTT intoxication most commonly occurs in patients with a history of chronic bronchitis.

### 3.5 Association of blood concentration of phenytoin with the prognosis of CPEHTT intoxication

Of the 104 patients with CPEHTT intoxication, blood concentration of phenytoin was detected in 92 patients. The blood concentration of phenytoin ranged between 14.5 and 94.12 μg/ml (the effective blood concentration is 10–20 μg/ml). A few patients had neurological symptoms at levels below 20 μg/ml, which may be related to liver and kidney function, drug sensitivity, and low body fat content. However, this does not affect the reference value of the effective blood concentration. Plasma concentration is an important indicator for monitoring phenytoin poisoning, but is not the only indicator in diagnosing poisoning. Therefore, for individualized treatment, the dosage of phenytoin should be adjusted in a timely manner to prevent poisoning based on blood concentration and clinical symptoms.

No special antidotes are available for phenytoin poisoning. Of the 104 patients with CPEHTT intoxication, most had improved symptoms after drug withdrawal, replacement with other antiasthmatic drugs, and treatment with folic acid, vitamins, complex coenzyme, and energy support. It has been reported that adverse reactions to CPEHTT were improved in 96% of cases after these treatments.^[1]^ Of the 6 severe adverse reactions, 3 were associated with anemia and hypoproteinemia and had poor clinical outcomes even after treatment. The plasma concentration of phenytoin after treatment was measured in 9 patients and ranged between 0.19 and 12 μg/ml. The average time for symptom improvement after treatment was 11 days (range, 4–30 days). Therefore, the treatment of CPEHTT poisoning is relatively simple, and the prognosis is very good.

## 4 Discussion

Since CPEHTT is a Chinese medical compound that is rarely used internationally, we only identified CPEHTT poisoning in China. We found 104 patients with CPEHTT intoxication in 10 published Chinese articles. We found that CPEHTT intoxication more commonly occurred in the elderly, with a male/female ratio of 2:1. All patients had a history of chronic lung disease, and the most common disease was chronic bronchitis, which required long-term or intermittent CPEHTT medication to reduce wheezing. Although CPEHTT is a prescribed drug, elderly patients often misused the drug due to a lack of guidance from professional physicians or pharmacists after initiation of treatment. Their medication compliance was poor, especially when the disease being treated was exacerbated. The neurological effects of CPEHTT toxicity are mainly caused by phenytoin.^[1]^ However, other ingredients of CPEHTT can also cause poisoning, and their clinical manifestations are different from phenytoin. For example, long-term use of ephedrine hydrochloride and caffeine can cause muscle tremors. Theophylline can excite the heart muscle and cause palpitations, convulsions, and a sharp drop in blood pressure.^[18]^ The anticholinergic adverse reactions of scopolamine can cause dysuria, constipation, dizziness, palpitations, dry mouth, and less sweat.^[19]^ In addition, the combination of these ingredients with phenytoin may aggravate the symptoms of adverse reactions. For instance, the combination of chlorpheniramine maleate and central nervous system inhibitory drugs can enhance its inhibitory effect, and chlorpheniramine maleate inhibits the metabolism of phenytoin via the liver microparticle enzyme.^[20]^ Therefore, phenytoin accumulation poisoning occurs when phenytoin and chlorpheniramine maleate are combined to.

As mentioned above, the factors related to CPEHTT intoxication mainly include age, sex, dosage, duration of medication use, and liver and kidney function. For elderly patients, the slow metabolism rate, poor liver and kidney function, and overdosing may contribute to CPEHTT poisoning. Since most of the CPEHTT patients are elderly men with chronic bronchitis, CPEHTT poisoning occurs more frequently in males than in females. In addition, most patients with CPEHTT poisoning did not take the recommended dose according to the instructions (Table 1). These patients may not know the risks of phenytoin or symptoms of overdose, and sometimes they increase the dosage to pursue better efficacy, which is very dangerous. At the same time, prolonged medication use is an important factor that contributes to poisoning. Therefore, clinicians and pharmacists should inform patients not to overuse drugs containing phenytoin, to be alert to possible poisoning symptoms, and to monitor blood levels of phenytoin regularly. The patients should be fully aware that drugs not only treat diseases, but also cause diseases if they are inappropriately used. The government and relevant health departments should improve education and supervision regarding drug safety. The pharmaceutical companies should inform patients about the possible risks of drug poisoning in instructions and improve public safety awareness. Since the precise diagnosis of most cases of CPEHTT poisoning requires a detailed medical history, clinicians should not ignore the importance of taking a medical history.

Long-term use and overdose of phenytoin sodium can induce acute and chronic poisoning. Therefore, CPEHTT intoxication may be associated with medication timing and dose. Consistent with this idea, we found that most patients had been taking excessive doses CPEHTT for a long time (>1 year). However, since some patients who took the recommended dose over a short time also had CPEHTT toxicity, further studies are required to demonstrate the association between CPEHTT toxicity and dose and duration.

In this review, we found that the common clinical manifestations of CPEHTT intoxication were dizziness, ataxia, limb weakness, diplopia, and binocular horizontal nystagmus. It has been reported that the primary symptoms of phenytoin toxicity are dizziness, nausea, poor appetite, and fatigue—as well as nystagmus, ataxia, dizziness and diplopia—due to damage to the cerebellum and labyrinthine pathway.^[21]^ The symptoms of CPEHTT toxicity are very similar to those of phenytoin toxicity, suggesting that phenytoin is the major culprit in CPEHTT intoxication. This is further supported by elevated plasma concentration of phenytoin sodium in patients with CPEHTT intoxication. Although it is known that phenytoin causes multiple system damage, the cerebellum is the most commonly affected site,^[22]^ resulting in clinical symptoms such as dizziness and ataxia. The symptoms of phenytoin poisoning are similar to those of cerebrovascular disease, and thus are often misdiagnosed as brain stem or cerebellar infarction^[23]^ Therefore, taking a detailed past medical history and CPEHTT medication history are important to avoid misdiagnosis, unnecessary imaging studies, and incorrect treatment.

The diagnosis of CPEHTT intoxication depends on the determination of phenytoin concentration. However, the normal range of the plasma phenytoin concentration may be associated with phenytoin poisoning. The history of medication use, clinical manifestations, and auxiliary examinations are also required to diagnose phenytoin poisoning.

For most patients with CPEHTT intoxication, the symptoms can significantly improve after drug withdrawal, replacement with other antiasthmatic drugs, and use of vitamins and energy support. For severe cases of CPEHTT intoxication, the symptoms improved after rehydration and symptomatic treatment. Poor prognosis may be associated with anemia and hypoproteinemia. The improvement of toxicity signs and symptoms is accompanied by a decrease in the plasma concentration of phenytoin.

There are some weaknesses and limitations in this study. First, this study included literatures with missing medical history and incomplete clinical data for some patients. The lack of the information prevents further analysis of the effect of these factors on phenytoin intoxication. Future studies are required to perform comprehensive analysis of CPEHTT intoxication in patients with complete clinical data. Second, although we found that age, sex, dosage, and medical duration are associated with CPEHTT intoxication, more persuasive causal association can not be analyzed due to incomplete data for some patients. Third, the sample size of this study is relatively small (n = 104 patients in 10 articles). A future multi-center study with a large sample size should be conducted to analyze the factors that affect CPEHTT intoxication.

In this review, we summarize the relevant literature on CPEHTT intoxication, which constitutes a drug safety problem in China. We discuss the common clinical manifestations, the underlying mechanism, and associated factors of CPEHTT intoxication. These findings should guide clinicians in the correct use of CPEHTT and reduce the risk of toxicity.

## Author contributions

**Conceptualization:** Lingxia Zhang.

**Data curation:** Mingyue Shan, Liping Chen.

**Formal analysis:** Mingyue Shan, Liping Chen.

**Funding acquisition:** Lingxia Zhang, Xu Han, Mingyue Shan, Liping Chen.

**Investigation:** Xu Han, Cancan Li.

**Methodology:** Zhenfei Li, Gaiying Ma, Cancan Li, Liping Chen.

**Project administration:** Gaiying Ma, Cancan Li.

**Software:** Zhenfei Li, Gaiying Ma.

**Supervision:** Zhenfei Li, Xu Han, Liping Chen.

**Writing – original draft:** Zhenfei Li, Liping Chen.

**Writing – review & editing:** Zhenfei Li, Liping Chen.
